# Normalization of Cardiac Function After Bariatric Surgery Is Related to Autonomic Function and Vitamin D

**DOI:** 10.1007/s11695-022-06336-x

**Published:** 2022-11-05

**Authors:** Sanne M. Snelder, Yaar Aga, Lotte E. de Groot - de Laat, L. Ulas Biter, Manuel Castro Cabezas, Nadine Pouw, Erwin Birnie, Bianca Boxma - de Klerk, René A. Klaassen, Felix Zijlstra, Bas M. van Dalen

**Affiliations:** 1 Department of Cardiology, Franciscus Gasthuis & Vlietland, Rotterdam, the Netherlands; 2 Department of Cardiology, Maasstad Ziekenhuis, Rotterdam, the Netherlands; 3 Department of Surgery, Franciscus Gasthuis & Vlietland, Rotterdam, the Netherlands; 4 Department of Internal Medicine, Franciscus Gasthuis & Vlietland, Rotterdam, the Netherlands; 5 Department of Clinical Chemistry, Franciscus Gasthuis & Vlietland, Rotterdam, the Netherlands; 6 Department of Statistics and Education, Franciscus Gasthuis & Vlietland, Rotterdam, the Netherlands; 7 Department of Surgery, Maasstad Ziekenhuis, Rotterdam, the Netherlands; 8 Department of Cardiology, the Thoraxcenter, Erasmus University Medical Centre, ’s Gravendijkwal 230, 3015 CE Rotterdam, the Netherlands; 9 Department of Internal Medicine, Erasmus University Medical Centre, ’s Gravendijkwal 230, 3015 CE Rotterdam, the Netherlands

**Keywords:** Obesity/obese, Bariatric surgery, Cardiac dysfunction, Global longitudinal strain, Autonomic dysfunction, Vitamin D

## Abstract

**Purpose:**

Subclinical cardiac dysfunction is common in patients with obesity. Bariatric surgery is associated with normalization of subclinical cardiac function in 50% of the patients with obesity. The aim of this study was to identify predictors for a lack of improvement of subclinical cardiac dysfunction 1-year post-bariatric surgery.

**Methods:**

Patients who were referred for bariatric surgery were enrolled in a longitudinal study. Inclusion criteria were age 35–65 years and BMI ≥ 35 kg/m^2^. Patients with a suspicion of or known cardiovascular disease were excluded. Conventional and advanced echocardiography, Holter monitoring, and blood tests were performed pre- and 1-year post-bariatric surgery. Subclinical cardiac dysfunction was defined as either a reduced left ventricular ejection fraction, decreased global longitudinal strain (GLS), diastolic dysfunction, arrhythmia, or an increased BNP or hs Troponin I.

**Results:**

A total of 99 patients were included of whom 59 patients had cardiac dysfunction at baseline. Seventy-two patients completed the 1-year follow-up after bariatric surgery. There was a significant reduction in weight and cardiovascular risk factors. Parameters of cardiac function, such as GLS, improved. However, in 20 patients cardiac dysfunction persisted. Multivariate analysis identified a decreased heart rate variability (which is a measure of autonomic function), and a decreased vitamin D pre-surgery as predictors for subclinical cardiac dysfunction after bariatric surgery.

**Conclusion:**

Although there was an overall improvement of cardiac function 1-year post-bariatric surgery, autonomic dysfunction and a decreased vitamin D pre-bariatric surgery were predictors for a lack of improvement of subclinical cardiac dysfunction.

**Graphical abstract:**

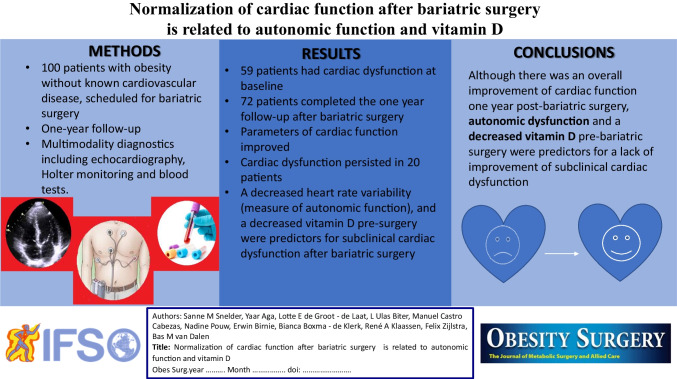

## Introduction

Obesity has reached epidemic proportions globally and the prevalence is still increasing [1]. Subclinical cardiac dysfunction is common in patients with obesity, [2] and obesity is associated with an increased risk of heart failure [3]. Heart failure is characterized by an impaired quality of life, frequent hospitalizations, and poor outcome [4]. Considering that prevention and treatment of heart failure have enormous medical and socioeconomic implications, a deeper understanding of risk factors for heart failure such as obesity is imperative.

Clinically significant weight loss is difficult to achieve with lifestyle interventions and the results are often temporary. In contrast, bariatric surgery is an effective and safe treatment option resulting in large long-term weight loss [5]. Several studies suggest that weight loss achieved by bariatric surgery has a positive impact on heart morphology, even in patients with obesity without heart failure [6]. We recently demonstrated that subclinical cardiac dysfunction normalized in half of the patients with obesity 1-year after bariatric surgery [7]. Also, bariatric surgery is associated with a 35% reduced incidence of new-onset heart failure during long term follow-up [8]. However, little is known about the pathophysiology of cardiac dysfunction in obesity patients and the factors determining the evolution of cardiac function after bariatric surgery are unknown. We have previously shown that subclinical cardiac dysfunction is related to autonomic dysfunction in obesity patients, [2] but it is unknown whether autonomic dysfunction may be related to a lack of recovery of cardiac dysfunction after bariatric surgery as well.

The CARdiac Dysfunction In Obesity – Early Signs Evaluation (CARDIOBESE) study was the first study in which (speckle tracking) echocardiography, blood tests, and Holter monitoring were combined to simultaneously investigate different aspects that may all play a role in the pathophysiology of subclinical cardiac dysfunction in obesity patients. The aim of this study was to identify predictors for persistent cardiac dysfunction 1-year post-bariatric surgery.

## Methods

### Study Design and Study Group

The protocol of the CARDIOBESE study has been described before [9]. In short, the CARDIOBESE study is a longitudinal study in which we prospectively enrolled 100 patients with obesity who were referred for bariatric surgery to the Franciscus Gasthuis & Vlietland (75 patients) and Maasstad Ziekenhuis (25 patients), both in Rotterdam, the Netherlands. Patients were included if they were between 35 and 65 years old and had a BMI of ≥ 35 kg/m [2]. Patients with a suspicion of or known cardiovascular disease were excluded. Bariatric surgery was performed by either a gastric sleeve, a gastric bypass or one anastomosis gastric bypass (OAGB) operation. Patients were seen pre- and 1-year post-bariatric surgery to study the intra-personal impact of obesity and bariatric surgery-related changes on cardiac function. The study protocol was approved by the ethics committee and written informed consent was obtained from all participants included in the study [9]. All procedures performed in studies involving human participants were in accordance with the ethical standards of the institutional and/or national research committee and with the 1964 Helsinki declaration and its later amendments or comparable ethical standards.

The presence or absence of subclinical cardiac dysfunction in the 100 patients with obesity of the CARDIOBESE-study has been described in detail before [2]. In short, cardiac dysfunction was defined as either a reduced LV ejection fraction, [10] a decreased global longitudinal strain (GLS) (< 17%), diastolic dysfunction [11], ventricular arrhythmia or an increased BNP (> 30 pmol/L) or hs Troponin I(≥ 34 ng/L for male and > 16 ng/L for female subjects). Of the predefined studied parameters, a decreased GLS (< 17%) was by far the most abundant, in 57 patients; one had diastolic dysfunction without an available GLS, one had a normal GLS but an increased BNP (49 pmol/L, normal value < 30 pmol/L), and one had a positive hs Troponin I. One patient with cardiac dysfunction was diagnosed with acromegaly after inclusion and was excluded from further analysis, leaving 59 patients with versus 40 without subclinical cardiac dysfunction.

### Transthoracic Echocardiography

Two-dimensional grayscale harmonic images were obtained in the left lateral decubitus position using a commercially available ultrasound system (EPIQ 7, Philips, Best, the Netherlands), equipped with a broadband (1-5 MHz) X5-1 transducer. All acquisitions and measurements were performed according to current guidelines. [10, 11]

Interventricular septal thickness (IVSd), posterior wall thickness (PWd), and left ventricular dimension (LVEDD) were all measured at end-diastole. The left ventricular mass (LVM) was calculated according to the Deveraux formula using these measurements: LVM (g) = 0.80 × {1.04[(IVSd + LVEDD + PWd)3-(LVEDD)3]} + 0.6. LVM index (LVMI) was calculated by dividing LVM by body surface area.

To optimize speckle tracking echocardiography, apical images were obtained at a frame rate of 60 to 80 frames/s. Three consecutive cardiac cycles were acquired from all apical views. Subsequently, these cycles were transferred to a QLAB workstation (version 10.2, Philips, Best, the Netherlands) for off-line speckle tracking analysis. Peak regional longitudinal strain was measured in 17 myocardial regions and a weighted mean was used to derive GLS.

### Blood Tests

Non-fasting blood samples were taken both for the study and as part of regular care. Routine laboratory measurements included; glucose, glycosylated haemoglobin (HbA1C), creatinine, estimated glomerular filtration rate (eGFR), alanine aminotransferase (ALAT), Apolipoprotein B, total cholesterol, low-density lipoprotein cholesterol (LDL-C), high-density lipoprotein cholesterol (HDL-C), triglycerides, ferritin, active vitamin B12, folic acid, vitamin B1, vitamin B6, albumin, magnesium, vitamin D, and haemoglobin were determined by standard clinical procedures as described [12]. In addition to the regular patient care path blood tests, high sensitive troponin I (hs troponin I), C reactive protein (CRP), and brain natriuretic peptide (BNP) were determined specifically for this study.

### Holter Monitoring

Heart rhythm was recorded for 24 consecutive hours using a portable digital recorder (GE HEER Light, USA). The digital recorder was connected using stickers that were placed on the chest. Average heart rate, minimal heart rate, maximum heart rate, total premature atrial contractions (PAC), total premature ventricular contractions (PVC), the standard deviation of all NN (often also referred to as RR) intervals (SDNN), and SDNN index were measured. A 24-h recording of the SDNN reveals the sympathetic nervous system contribution to heart rate variability [13]. The SDNN index estimates the variability due to the factors affecting heart rate variability (HRV) within a 5-min period. It is calculated by first dividing the 24-h record into 288 5-min-segments and then calculating the standard deviation of all NN intervals contained within each segment. [14]

### Statistical Analysis

Patients who completed the follow-up were included in the analysis. The normality of the data was checked by the Shapiro–Wilk test. Continuous values with normal distributions were expressed as mean ± standard deviation, with skewed distributions as median and interquartile range and categorical values as percentages. The paired Student’s *t*-test was used for continuous variables with normal distributions, the nonparametric Wilcoxon signed-rank test for variables with skewed distributions, and the McNemar test for categorical variables was used to compare parameters pre- and post-surgery.

The unpaired Student’s *t*-test for continuous variables was used to compare the pre- and post-surgery values of patients with versus without cardiac dysfunction post-surgery, the non-parametric Mann–Whitney *U* test for continuous parameters with skewed distributions, and the *χ*^2^ test for categorical variables. Pre-surgery parameters that significantly differed between patients with post-surgery normal cardiac function and patients with post-surgery cardiac dysfunction in the univariate analyses were added to multivariate logistic regression analysis (method: backward stepwise analysis). The discriminative ability of the resulting model was investigated by calculating the area under the receiver operating curve (AUC). Odds ratios and 95% confidence intervals were calculated. A two-tailed *p*-value of < 0.05 was considered statistically significant. Statistical analyses were performed with SPSS version 26.0 or higher (SPSS Inc., Chicago, USA).

## Results

### Changes in Features of Obesity from Pre- to 1-Year Post-Bariatric Surgery

**Fig. 1:**
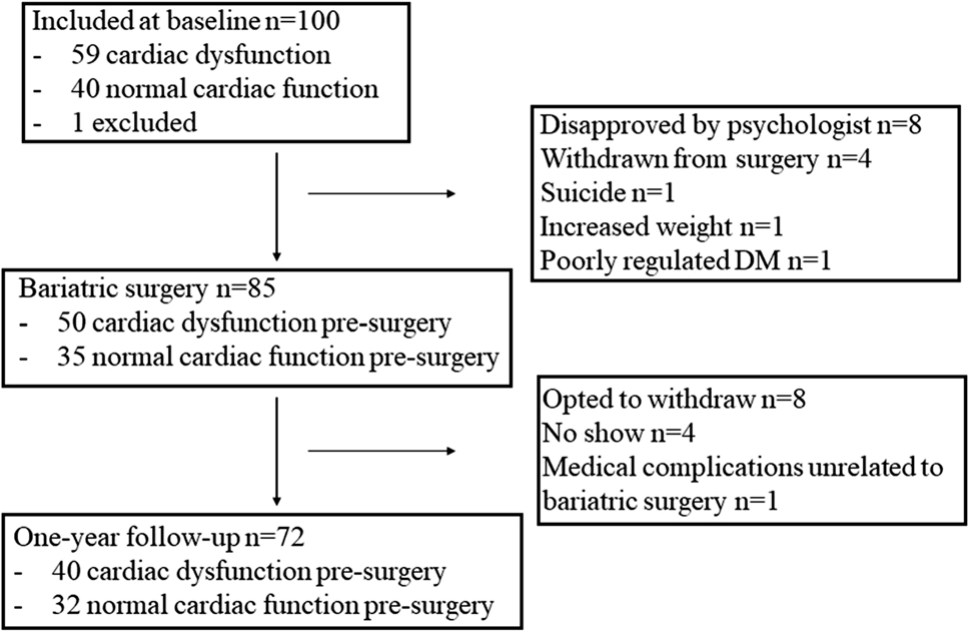
Flow-chart of patients with completion of or loss to follow-up. DM = diabetes mellitus

A total of 100 patients with obesity were included, 85 patients underwent bariatric surgery and 72 patients completed the 1-year follow-up (Fig. 1). Fifteen patients did not undergo bariatric surgery because of various reasons, but mostly because of disapproval by the psychologist or because they withdrew from surgery for personal reasons.

In Table 1, it is shown that weight loss and decreased BMI were significant 1-year post-bariatric surgery. Systolic blood pressure and heart rate decreased significantly as well. Also, the prevalence of comorbidities such as diabetes mellitus, hypertension, and obstructive sleep apnoea syndrome decreased significantly. Medication use was reduced post-surgery, with a significant reduction in use of ACE inhibitors/angiotensin receptor blockers, statins, and oral anti-diabetics.

**Table 1:** Clinical characteristics of the study population. Differences between obesity patients from pre- to 1-year post-bariatric surgery

	Pre-surgery (*n* = 72)	1-year post-surgery (*n* = 72)	*p*-value
General characteristics			
Age (years)	48 (43–54)		
Female (*n*, %)	54 (75%)		
Physical examination			
Weight (kg)	122 [113–133]	83 [74–91]	< 0.001
BMI (kg/m^2^)	41 [39–46]	28 [25–31]	< 0.001
Systolic BP (mmHg)	146 ± 21	133 ± 20	0.003
Diastolic BP (mmHg)	79 [73–88]	80 [75–86]	0.18
Heart rate (bpm)	80 [73–86]	65 [57–71]	< 0.001
Comorbidity			
Diabetes mellitus (*n*, %)	16 (22%)	6 (8%)	0.002
Hypertension (*n*, %)	24 (33%)	12 (17%)	0.035
Hypercholesterolemia (*n*, %)	15 (21%)	8 (11%)	0.09
Current smoking (*n*, %)	11 (15%)	3 (6%)	0.18
COPD (*n*, %)	4 (6%)	0	0.13
OSAS (*n*, %)	8 (11%)	0	0.008
Medication			
Beta blockers (*n*, %)	5 (7%)	3 (4%)	0.63
ACE inhibitors/ARBs (*n*, %)	11 (15%)	8 (11%)	0.012
Calcium channel blockers (*n*, %)	6 (8%)	5 (7%)	0.66
Statins (*n*, %)	16 (22%)	9 (13%)	0.039
Diuretics (*n*, %)	13 (18%)	8 (11%)	0.18
Insulin (*n*, %)	5 (7%)	4 (6%)	0.56
Oral anti-diabetics (*n*, %)	10 (14%)	4 (6%)	0.031
Blood tests			
CRP (mg/L)	6 [3–9]	0 [0–2]	< 0.001
Glucose (mmol/L)	5.4 [4.8–6.4]	5.0 [4.6–5.6]	0.051
HbA1c (mmol/mol)	39 [35–48]	36 [33–39]	< 0.001
Creatinine(umol/L)	70 [65–78]	67 [62–71]	< 0.001
eGFR (ml/min/1.73m^2^)	83 ± 9	87 ± 5	< 0.001
ALAT (U/L)	30 [20–37]	19 [15–26]	0.004
Apolipoprotein B (g/L)	1.04 [0.88–1.25]	0.84 [0.73–1.05]	< 0.001
Total cholesterol (mmol/L)	5.3 ± 0.9	4.6 ± 0.8	< 0.001
LDL cholesterol (mmol/L)	3.2 ± 0.8	2.6 ± 0.7	< 0.001
HDL cholesterol (mmol/L)	1.2 [1.0–1.4]	1.4 [1.2–1.6]	< 0.001
Triglycerides (mmol/L)	1.7 [1.3–2.3]	1.0 [0.8–1.4]	< 0.001
Ferritin (ug/L)	83 [53–177]	97 [49–171]	0.60
Active Vitamin B12 (pmol/L)	101 [71–132]	104 [66–128]	0.24
Folic acid (nmol/L)	13 [9–16]	27 [16–36]	< 0.001
Vitamin B1 (nmol/L)	140 ± 28	131 ± 40	0.17
Vitamin B6 (nmol/L)	67 [52–81]	98 [61–128]	0.009
Albumin (g/L)	42 [39–44]	41 [40–43]	0.033
Magnesium (mmol/L)	0.82 [0.76–0.87]	0.82 [0.78–0.86]	0.38
Vitamin D (nmol/L)	39 [27–66]	75 [61–98]	< 0.001
Haemoglobin (mmol/L)	8.8 [8.1–9.1]	8.5 [8.0–9.1]	0.012
Echocardiography parameters			
Left ventricular mass (g)	177 [138–214]	150 [121–182]	< 0.001
LVM index (g/m^2^)	72 [59–87]	77 [64–87]	0.49
Holter monitoring			
Ventricular arrhythmia (*n*, %)	0	0	
Average heart rate (bpm)	83 ± 10	73 ± 8	< 0.001
Minimal heart rate (bpm)	53 [47–57]	46 [44–51]	< 0.001
Maximum heart rate (bpm)	137 [128–150]	130 [120–142]	0.005
SDNN (ms)	106 ± 46	124 ± 47	< 0.001
SDNN index (ms)	46 [38–57]	59 [49–69]	< 0.001

Values represent mean ± SD, median (Q1–Q3), or *n* (%)

*p*-values displayed were analysed by the paired Student’s *t*-test for continuous variables with normal distributions, the nonparametric Wilcoxon signed-rank test for variables with skewed distributions, and the McNemar test for categorical variables

*BMI*, body mass index; *BP*, blood pressure; *COPD*, chronic obstructive pulmonary disease; *OSAS*, obstructive sleep apnoea syndrome; *ACE*, angiotensin-converting enzyme; *ARBs*, angiotensin II receptor blockers; *CRP*, C-reactive protein; *HbA1c*, glycated haemoglobin; *eGFR*, estimated glomerular filtration rate; *ALAT*, alanine transaminase; *LDL*, low-density lipoprotein; *HDL*, high-density lipoprotein; *LVM index*, left ventricular mass index; *SDNN*, standard deviation of NN intervals; *SDNN index*, mean of the standard deviations of all the NN intervals for each 5-min segment of a 24-h heart rate variability recording

Blood tests showed a significant decrease in CRP, HbA1c, creatinine, ALAT, Apolipoprotein B, total cholesterol, LDL-C, and triglycerides post-bariatric surgery. HDL-C, folic acid, vitamin B6, and vitamin D increased significantly. The echocardiogram showed a decrease in LVM, but when corrected for the body surface area (LVM index), there was no significant decrease. Holter monitoring showed a decreased mean and minimal and maximum heart rate 1-year post-surgery, whereas the SDNN and the SDNN index increased.

### Changes of Parameters of Cardiac Dysfunction from Pre- to 1-Year Post-Bariatric Surgery

There was a mild but statistically significant increase in BNP 1-year post-bariatric surgery (Table 2). Levels of hs troponin I were comparable. Echocardiography showed a significant improvement of GLS. The prevalence of diastolic dysfunction and the LV ejection fraction did not change. Also, the frequency of extrasystoles did not change from pre- to 1-year post-bariatric surgery.

**Table 2:** Parameters of cardiac function. Differences between obesity patients from pre- to 1-year post-bariatric surgery

	Pre-surgery (*n* = 72)	1-year post-surgery (*n* = 72)	*p*-value
Blood tests			
BNP (pmol/L)	5 [3–8]	8 [6–10]	**0.029**
hs troponin I positive (*n*, %)	1 (1%)	5 (7%)	0.06
Echocardiography parameters			
Mitral inflow E-wave (cm/s)	66 ± 16	69 ± 14	0.45
Mitral inflow A-wave (cm/s)	71 ± 14	65 ± 12	** < 0.001**
*E*/*A* ratio	0.98 [0.9–1.1]	1.1 [0.9–1.2]	**0.008**
Septal *e*′ velocity (cm/s)	7.8 ± 2.1	8.3 ± 1.7	0.56
Lateral *e*′ velocity (cm/s)	9.6 ± 3.1	12.2 ± 3.1	** < 0.001**
*E*/*e*′ ratio	8.7 [7.5–9.9]	8.3 [7.0–9.6]	0.07
Deceleration time (s)	0.18 [0.17–0.21]	0.18 [0.15–0.21]	0.51
LA volume index (ml/m^2^)	24 [20–31]	27 [23–34]	0.07
TR velocity (cm/s)	106 [91–139]	191 [106–218]	** < 0.001**
Diastolic dysfunction (*n*, %)	7 (10%)	3 (4%)	0.28
LV ejection fraction (%)	58 ± 8	57 ± 7	0.25
Global longitudinal strain (%)	− 15.6 ± 3.1	− 18.1 ± 3.3	**0.001**
Holter monitoring			
Total PAC per 24 h (*n*)	9 [2–38]	20 [8–68]	0.07
Total PVC per 24 h (*n*)	3 [0–22]	5 [2–58]	0.29
Supraventricular arrhythmia (*n*, %)	1 (1%)	0	0.53
Ventricular arrhythmia (*n*, %)	0	0	

Values represent mean ± SD, median (Q1–Q3), or *n* (%)

*p*-values displayed were analysed by the paired Student’s *t*-test for continuous variables with normal distributions, the nonparametric Wilcoxon signed-rank test for variables with skewed distributions, and the McNemar test for categorical variables

*BNP*, brain natriuretic peptide; *hs troponin I*, high sensitive troponin I; *E-wave*, early diastolic transmitralflow velocity; *A-wave*, late diastolic transmitralflow velocity; *e*′, early diastolic mitral annular velocity; *LA volume index*, left atrial volume index; *TR velocity*, tricuspid regurgitation; *LV*, left ventricular; *PAC*, premature atrial contraction; *PVC*, premature ventricular contraction

### Comparison of Patients with Versus Without Normalization of Cardiac Function After Bariatric Surgery

Of the patients with complete follow-up, 40 (56%) had subclinical cardiac dysfunction pre-surgery. In 50% of these patients, cardiac function had normalized 1-year post-surgery (Table 3). In the 20 patients in whom subclinical cardiac dysfunction persisted, 17 (43%) had a decreased GLS, one patient had an elevated hs troponin I level, and two patients had diastolic dysfunction.

**Table 3:** Comparison of characteristics of patients with pre-existent cardiac dysfunction subdivided into those who showed normalization of cardiac function after bariatric surgery compared to those with persistent cardiac dysfunction

	Post-surgery normal cardiac function (*n* = 20)		Post-surgery cardiac dysfunction (*n* = 20)		*p*-value pre	*p*-value post
	Pre-surgery	Post-surgery	Pre-surgery	Post-surgery		
General characteristics						
Age (years)	48 ± 7		51 ± 8		0.19	
Female (*n*, %)	13 (65%)		12 (60%)		0.74	
Physical examination						
Weight (kg)	121 [113–132]	83 [75–90]	125 [111–144]	84 [76–98]	0.37	0.62
BMI (kg/m^2^)	41 [40–46]	28 [26–31]	42 [39–46]	28 [26-30]	0.83	0.76
Systolic BP (mmHg)	140 [130–159]	138 [116–148]	147 [137–160]	128 [121–134]	0.34	0.48
Diastolic BP (mmHg)	80 ± 13	78 ± 10	86 ± 14	81 ± 7	0.23	0.54
Heart rate (bpm)	80 [78–93]	67 [59–73]	80 [77–88]	63 [53–73]	0.41	0.40
Comorbidity						
Diabetes mellitus (*n*, %)	6 (30%)	2 (10%)	4 (20%)	2 (10%)	0.46	1
Hypertension (*n*, %)	9 (45%)	3 (15%)	7 (35%)	4 (20%)	0.52	0.62
Hypercholesterolemia (*n*, %)	7 (35%)	3 (15%)	3 (15%)	4 (20%)	0.14	0.62
Current smoking (*n*, %)	1 (5%)	2 (10%)	3 (15%)	1 (5%)	0.29	0.74
COPD (*n*, %)	1 (5%)	0	0	0	0.31	
OSAS (*n*, %)	3 (15%)	0	3 (15%)	0	1	
Medication						
Beta blockers (*n*, %)	3 (15%)	2 (10%)	0	1 (5%)	0.07	0.72
ACE inhibitors/ARBs (*n*, %)	5 (25%)	2 (10%)	5 (25%)	3 (15%)	1	0.54
Calcium channel blockers (*n*, %)	3 (15%)	1 (5%)	2 (10%)	2 (10%)	0.63	0.49
Statins (*n*, %)	8 (40%)	3 (15%)	4 (20%)	5 (25%)	0.17	0.34
Diuretics (*n*, %)	5 (25%)	2 (10%)	3 (15%)	33 (15%)	0.43	0.54
Insulin (*n*, %)	3 (15%)	2 (10%)	1 (5%)	1 (5%)	0.29	0.62
Oral anti-diabetics (*n*, %)	4 (20%)	1 (5%)	2 (10%)	1 (5%)	0.38	0.94
Blood tests						
BNP (pmol/L)	5 [3–6]	7 [4–11]	3 [3–7]	8 [6–11]	0.72	0.83
hs Troponin I positive (*n*)	0	0	0	2 (10%)		0.15
CRP (mg/L)	5 [4–9]	1 [0–3]	6 [4–9]	0 [0–1]	0.64	0.38
Glucose (mmol/L)	6.4 ± 2.2	5.6 ± 1.6	7.2 ± 3.3	6.5 ± 2.2	0.37	0.23
HbA1c (mmol/mol)	51 ± 18	40 ± 9	44 ± 12	38 ± 3	0.13	0.41
Creatinine(umol/L)	71 [65–78]	68 [60–71]	71 [63–77]	66 [64–73]	0.94	0.74
eGFR (ml/min/1.73m^2^)	85 ± 8	87 ± 5	85 ± 9	89 ± 3	0.93	0.60
ALAT (U/L)	31 [21–51]	19 [16–29]	31 [27–37]	18 [14–26]	0.91	0.39
Apolipoprotein B (g/L)	0.98 ± 0.26	0.92 ± 0.22	1.1 ± 0.28	0.89 ± 0.22	0.22	0.84
Total cholesterol (mmol/L)	5.0 ± 1.0	4.6 ± 0.7	5.2 ± 0.9	4.6 ± 0.8	0.53	0.89
LDL cholesterol (mmol/L)	2.8 ± 0.6	2.7 ± 0.7	3.0 ± 0.8	2.6 ± 0.9	0.59	0.62
HDL cholesterol (mmol/L)	1.1 [1.0–1.3]	1.3 [1.1–1.4]	1.1 [1.0–1.3]	1.4 [1.2–1.7]	0.98	0.24
Triglycerides (mmol/L)	2.2 ± 1.4	1.3 ± 0.7	2.3 ± 1.1	1.5 ± 0.9	0.74	0.56
Ferritin (ug/L)	150 ± 142	128 ± 90	134 ± 70	153 ± 139	0.66	0.53
Active Vitamin B12 (pmol/L)	82 [70–114]	95 [62–128]	97 [60–108]	128 [74–303]	0.83	0.06
Folic acid (nmol/L)	13 [11–17]	28 [16–35]	13 [9–17]	25 [10–45]	0.60	0.79
Vitamin B1 (nmol/L)	150 ± 24	147 ± 55	149 ± 21	133 ± 34	0.93	0.52
Vitamin B6 (nmol/L)	95 ± 88	112 ± 39	69 ± 17	82 ± 26	0.39	0.06
Albumin (g/L)	43 ± 3	42 ± 3	39 ± 3	40 ± 3	0.002	0.008
Magnesium (mmol/L)	0.83 ± 0.05	0.84 ± 0.05	0.81 ± 0.05	0.82 ± 0.04	0.43	0.45
Vitamin D (nmol/L)	54 [30–80]	80 [67–98]	33 [25–54]	62 [42–104]	0.04	0.12
Haemoglobin (mmol/L)	8.9 ± 0.5	8.8 ± 0.6	8.7 ± 0.8	8.8 ± 1.0	0.42	0.91
Echocardiography parameters						
Mitral inflow E-wave (cm/s)	68.8 ± 10.6	69.5 ± 16.3	64.1 ± 8.7	62.8 ± 12.0	0.14	0.15
Mitral inflow A-wave (cm/s)	69.1 ± 12.0	66.2 ± 12.0	72.8 ± 15.1	63.7 ± 10.0	0.45	0.48
*E*/*A* ratio	0.97 [0.92–1.00]	0.94 [0.80–1.35]	0.88 [0.76–1.01]	1.05 [0.85–1.10]	0.29	0.98
Septal *e*′ velocity (cm/s)	8.1 ± 1.6	8.3 ± 2.1	7.8 ± 1.9	7.9 ± 1.4	0.64	0.54
Lateral *e*′ velocity (cm/s)	10.5 ± 2.3	12.2 ± 3.6	9.8 ± 3.0	10.9 ± 2.4	0.45	0.20
*E*/*e*′ ratio	8.7 [7.6–9.7]	8.5 [7.6–9.7]	8.0 [6.8–9.9]	7.9 [6.6–9.3]	0.45	0.42
Deceleration time (s)	0.19 ± 0.03	0.20 ± 0.05	0.19 ± 0.04	0.19 ± 0.05	0.60	0.49
LA volume index (ml/m2)	24.7 ± 7.6	28.7 ± 8.3	26.4 ± 9.7	27.7 ± 6.3	0.56	0.70
TR velocity (cm/s)	92 [90–169]	180 [101–214]	97 [82–112]	189 [111–214]	0.81	0.42
Left ventricular mass (g)	179 [140–226]	157 [132–196]	202 [140–235]	151 [128–200]	0.43	0.70
LVM index (g/m^2^)	72 [61–89]	81 [69–97]	78 [62–92]	76 [65–90]	0.53	0.27
Holter monitoring						
Total PAC per 24 h (*n*)	7 [2–41]	24 [9–107]	15 [2–56]	19 [9–90]	0.44	0.63
Total PVC per 24 h (*n*)	3 [0–18]	4 [2–30]	4 [0–32]	4 [1–89]	0.51	0.86
Average heart rate (bpm)	86 ± 8	75 ± 7	82 ± 11	74 ± 7	0.21	0.68
Minimal heart rate (bpm)	57 ± 13	48 ± 5	51 ± 7	45 ± 10	0.08	0.18
Maximum heart rate (bpm)	136 ± 15	135 ± 18	138 ± 14	125 ± 33	0.58	0.26
SDNN (ms)	107 [77–136]	145 [117–155]	77 [46–98]	84 [65–160]	0.011	0.09
SDNN index (ms)	45 ± 15	58 ± 15	42 ± 6	59 ± 22	0.64	0.98

Values represent mean ± SD, median (Q1–Q3), or *n* (%)

*p*-value pre and *p*-value post represent comparison of pre- and post-surgery values respectively. *p*-values displayed were analysed with the unpaired Student’s *t*-test for continuous variables, the non-parametric Mann–Whitney *U* test for continuous parameters with skewed distributions, and the *χ*^2^ test for categorical variables

*BMI*, body mass index; *BP*, blood pressure; *COPD*, chronic obstructive pulmonary disease; OSAS, obstructive sleep apnoea syndrome; *ACE*, angiotensin-converting enzyme; *ARBs*, angiotensin II receptor blockers; *BNP*, brain natriuretic peptide; hs troponin I, high sensitive troponin I; *CRP*, C-reactive protein; *HbA1c*, glycated haemoglobin; *eGFR*, estimated glomerular filtration rate; *ALAT*, alanine transaminase; *LDL*, low-density lipoprotein; *HDL*, high-density lipoprotein; *E-wave*, early diastolic transmitralflow velocity; *A-wave*, late diastolic transmitralflow velocity; *e*′, early diastolic mitral annular velocity; *LA volume index*, left atrial volume index; *TR velocity*, tricuspid regurgitation; *LVM index*, left ventricular mass index; *PAC*, premature atrial contraction; *PVC*,, premature ventricular contraction; *SDNN*, standard deviation of NN intervals; *SDNN index*, mean of the standard deviations of all the NN intervals for each 5-min segment of a 24-h heart rate variability recording

**Fig. 2:**
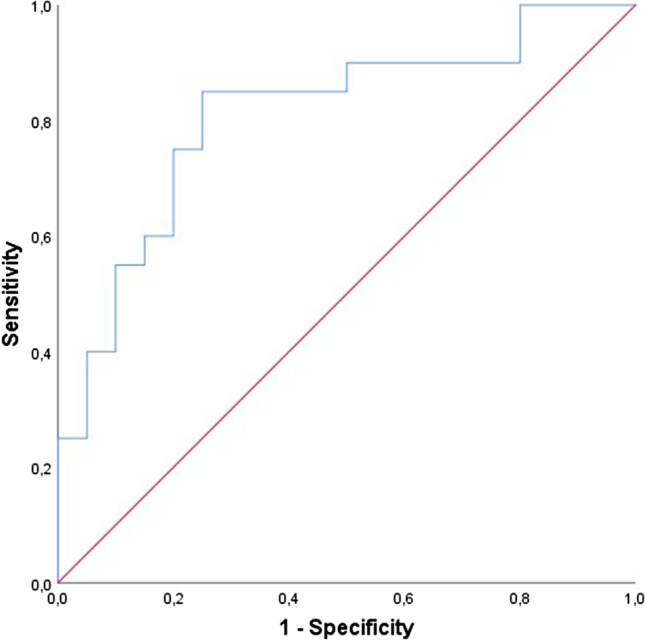
ROC-curve for the prediction model for cardiac dysfunction post-surgery. Model; combination of SDNN and vitamin D pre-surgery. Area under the curve = 0.81 (95% CI: 0.67–0.95, *p* = 0.001), sensitivity of 70%, and a specificity of 80%

When comparing patients with versus without normalization of cardiac function after bariatric surgery, most pre-surgery parameters were comparable, except for albumin, vitamin D, and SDNN. Post-surgery only albumin was mildly decreased in patients without normalization. Multivariate analysis was applied including the parameters which were different pre-surgery, identifying a decreased SDNN and a decreased vitamin D pre-surgery as significant predictors for maintaining cardiac dysfunction after bariatric surgery (Table 4). The multivariate model including these two parameters to identify patients who maintained cardiac dysfunction post-surgery had an AUC of 0.81 (95% CI: 0.67–0.95, *p* = 0.001), with a sensitivity of 70% (95%CI: 66–87%) and a specificity of 80% (95%CI: 56–93%) (Fig. 2).

**Table 4:** Univariable and multivariable logistic regression analysis in obesity patients with pre-surgery cardiac dysfunction, with presence of subclinical cardiac dysfunction post-surgery as the dependent variable

Variable	Univariate analysis		Multivariable analysis	
	OR (95% CI)	*p*-value	OR (95% CI)	*p*-value
SDNN	0.97 (0.96–1.00)	0.015	0.98 (0.96–1.00)	0.014
Vitamin D	0.97 (0.54–1.00)	0.048	0.97 (0.94–1.00)	0.043
Albumin	0.73 (0.58–0.92)	0.006		

Variables displayed were statistically significant different between obesity patients with and without cardiac dysfunction. Multivariable logistic regression analysis; method: backward stepwise analysis

*OR*, odds ratio; *95% CI*, 95% confidence interval; *SDNN*, standard deviation of NN intervals

## Discussion

The main finding of the current study is that persistence of cardiac dysfunction in patients with obesity 1 year after bariatric surgery was related to autonomic dysfunction and a decreased vitamin D pre-surgery.

Although in previous studies changes of cardiac morphology and function after bariatric surgery have been investigated, [6, 8] CARDIOBESE is the first study in which the focus was specifically on *subclinical* cardiac dysfunction. Furthermore, analysis with the combination of speckle tracking echocardiography, blood tests, and Holter monitoring was used for the first time to simultaneously investigate different aspects of cardiac dysfunction and the underlying pathophysiology. As expected, and in-line with previous findings, [6, 8] many cardiovascular risk factors and parameters of cardiac function improved post-surgery. Prevalence of comorbidities decreased, lipid levels and HbA1c improved, and CRP decreased. Also, there was a mild but statistically significant increase of BNP 1-year post-surgery. BNP is known to be decreased in patients with obesity, both with and without heart failure [15]. Although the reason for this remains incompletely understood, it is most likely due to lower release in these patients, rather than increase in their clearance. [16]

Improvement of LV function following bariatric surgery has been described before in small studies [17–20]. However, CARDIOBESE is the largest study in which speckle tracking echocardiography was used to investigate improvement of LV function after bariatric surgery. As we recently reported, there was an overall improvement of GLS 1-year post-surgery, resulting in normalization of subclinical cardiac dysfunction in 50% of the patients with obesity. [7]

While it was already known that autonomic dysfunction as expressed by a decreased HRV may be related to either cardiac dysfunction [21] or to obesity, [22] previously reported baseline data of the patients included in the CARDIOBESE study [2] for the first time showed that autonomic dysfunction appears to have a prominent role in the pathophysiology of cardiac dysfunction in obesity. However, so far, it was unknown whether autonomic dysfunction may play a role in *persistence* of cardiac dysfunction after bariatric surgery as well. In the current study, it was shown that a decreased SDNN pre-surgery was a predictor for persistent subclinical cardiac dysfunction 1-year post-bariatric surgery. The SDNN represents the beat-to-beat variation during Holter monitoring by measuring the standard deviation of NN intervals [22]. The SDNN is a parameter of autonomic function through the sympathetic nervous system contribution to HRV [13]. A balanced autonomic function is crucial for normal cardiac function [21]. On the other hand, a depressed HRV is related to morbidity and mortality [23, 24]. Oher studies already described a favourable effect of bariatric surgery on HRV [25]. Yet, by combining findings from Holter monitoring and echocardiography, our study is the first to relate the severity of autonomic dysfunction in obesity to the potential of recovery of cardiac dysfunction after bariatric surgery.

In the patients in our study, there was a significant increase in SDNN 1-year post-surgery, indicative of improvement of autonomic function, both in patients with improvement of LV function and in patients with persistent LV dysfunction. It can therefore be hypothesized that more severe autonomic dysfunction in obesity as expressed by decreased SDNN pre-surgery, may lead to either a permanent or delayed lack of improvement of LV function after bariatric surgery. Longer follow-up of obesity patient post-bariatric surgery may elucidate whether LV function will improve after all, in-line with improvement of autonomic function.

While as described above, a role of autonomic dysfunction was somewhat anticipated, the finding that a decreased vitamin D before bariatric surgery was also independently related to persistent subclinical cardiac dysfunction 1-year post-surgery was less expected. Nevertheless, vitamin D has been suggested to be involved in multiple pathophysiological pathways related to heart failure, such as inflammation, atherosclerosis, endothelial dysfunction, and thrombosis [26]. Furthermore, vitamin D deficiency is a predictor of reduced survival in patients with heart failure [27]. Also, vitamin D is known to be decreased in patients with obesity, [28] and in patients with known cardiovascular disease [29], suggesting that vitamin D may have a role in the increased risk of cardiac dysfunction in obesity. However, previous studies from our group failed to show significant effects of vitamin D supplementation on inflammatory changes in females with overweight, making this mechanism less likely [30]. Although the underlying mechanism remains to be elucidated, by combining findings from blood tests and echocardiography in our study, it was shown for the first time that a relative decreased vitamin D level pre-bariatric surgery is related to a lack of improvement of cardiac function after bariatric surgery.

### Limitations

A relatively large number (32%) of the patients with cardiac dysfunction did not complete the follow-up: 15% because they did not undergo bariatric surgery, and 17% dropped out because of various other reasons. Meanwhile, 20% of the patients with a normal cardiac function was lost to follow-up. The reason for this difference is unknown, but probably it was just coincidence. Furthermore, follow-up after bariatric surgery was 1 year and it may be hypothesized that a longer follow-up would have shown improvement of cardiac function in a larger proportion of patients.

## Conclusions

Autonomic dysfunction at baseline was related to a lack of normalization of cardiac function in patients with obesity 1 year after bariatric surgery. This result is in-line with previous findings of our group, [2] confirming an important role of autonomic dysfunction in the pathophysiology of cardiac dysfunction in obesity. Decreased vitamin D before bariatric surgery was also independently related to persistent subclinical cardiac dysfunction 1-year post-surgery. Since this finding was less expected, we consider this less affirmative and more hypothesis-generating. Nevertheless, signs of either autonomic dysfunction or a decreased vitamin D pre-bariatric surgery may be indicative of a need for cardiologic follow-up after bariatric surgery.

## References

[1] Global BMIMC, Di Angelantonio E, Bhupathiraju ShN, Wormser D, Gao P, Kaptoge S (2016). Body-mass index and all-cause mortality: individual-participant-data meta-analysis of 239 prospective studies in four continents. Lancet.

[2] Snelder SM, de Groot-de Laat LE, Biter LU, Castro Cabezas M, Pouw N, Birnie E (2020). Subclinical cardiac dysfunction in obesity patients is linked to autonomic dysfunction: findings from the CARDIOBESE study. ESC Heart Fail.

[3] Kenchaiah S, Evans JC, Levy D, Wilson PW, Benjamin EJ, Larson MG (2002). Obesity and the risk of heart failure. N Engl J Med.

[4] Stewart S, MacIntyre K, Hole DJ, Capewell S, McMurray JJ (2001). More 'malignant' than cancer? Five-year survival following a first admission for heart failure. Eur J Heart Fail.

[5] Sjostrom L, Lindroos AK, Peltonen M, Torgerson J, Bouchard C, Carlsson B (2004). Lifestyle, diabetes, and cardiovascular risk factors 10 years after bariatric surgery. N Engl J Med.

[6] Vest AR, Heneghan HM, Agarwal S, Schauer PR, Young JB (2012). Bariatric surgery and cardiovascular outcomes: a systematic review. Heart.

[7] Snelder SM, Aga Y, Laat LEdG-d, Biter LU, Cabezas MC, Pouw N, et al. Cardiac function normalizes one year after bariatric surgery in half of the obesity patients with subclinical cardiac dysfunction. Obes Surg. 2021;31(9):4206–09. 10.1007/s11695-021-05423-9 33884567

[8] Jamaly S, Carlsson L, Peltonen M, Jacobson P, Karason K (2019). Surgical obesity treatment and the risk of heart failure. Eur Heart J..

[9] Snelder SM, de Groot-de Laat LE, Biter LU, Castro Cabezas M, van de Geijn GJ, Birnie E (2018). Cross-sectional and prospective follow-up study to detect early signs of cardiac dysfunction in obesity: protocol of the CARDIOBESE study. BMJ Open.

[10] Lang RM, Badano LP, Mor-Avi V, Afilalo J, Armstrong A, Ernande L (2015). Recommendations for cardiac chamber quantification by echocardiography in adults: an update from the American Society of Echocardiography and the European Association of Cardiovascular Imaging. Eur Heart J Cardiovasc Imaging.

[11] Nagueh SF, Smiseth OA, Appleton CP, Byrd BF, Dokainish H, Edvardsen T (2016). Recommendations for the evaluation of left ventricular diastolic function by echocardiography: an update from the American Society of Echocardiography and the European Association of Cardiovascular Imaging. Eur Heart J Cardiovasc Imaging.

[12] van Mil SR, Biter LU, van de Geijn GM, Birnie E, Dunkelgrun M, JNM IJ (2018). Contribution of type 2 diabetes mellitus to subclinical atherosclerosis in subjects with morbid obesity. Obes Surg.

[13] Grant CC, van Rensburg DC, Strydom N, Viljoen M (2011). Importance of tachogram length and period of recording during noninvasive investigation of the autonomic nervous system. Ann Noninvasive Electrocardiol.

[14] Shaffer F, Ginsberg JP (2017). An overview of heart rate variability metrics and norms. Front Public Health.

[15] Mehra MR, Uber PA, Park MH, Scott RL, Ventura HO, Harris BC (2004). Obesity and suppressed B-type natriuretic peptide levels in heart failure. J Am Coll Cardiol.

[16] Mueller C, McDonald K, de Boer RA, Maisel A, Cleland JGF, Kozhuharov N (2019). Heart Failure Association of the European Society of Cardiology practical guidance on the use of natriuretic peptide concentrations. Eur J Heart Fail.

[17] Leung M, Xie M, Durmush E, Leung DY, Wong VW (2016). Weight loss with sleeve gastrectomy in obese type 2 diabetes mellitus: impact on cardiac function. Obes Surg.

[18] Di Bello V, Santini F, Di Cori A, Pucci A, Talini E, Palagi C (2008). Effects of bariatric surgery on early myocardial alterations in adult severely obese subjects. Cardiology.

[19] Koshino Y, Villarraga HR, Somers VK, Miranda WR, Garza CA, Hsiao JF (2013). Changes in myocardial mechanics in patients with obesity following major weight loss after bariatric surgery. Obesity (Silver Spring).

[20] Shin SH, Lee YJ, Heo YS, Park SD, Kwon SW, Woo SI (2017). Beneficial effects of bariatric surgery on cardiac structure and function in obesity. Obes Surg.

[21] Florea VG, Cohn JN (2014). The autonomic nervous system and heart failure. Circ Res.

[22] Yadav RL, Yadav PK, Yadav LK, Agrawal K, Sah SK, Islam MN (2017). Association between obesity and heart rate variability indices: an intuition toward cardiac autonomic alteration - a risk of CVD. Diabetes Metab Syndr Obes.

[23] Forslund L, Bjorkander I, Ericson M, Held C, Kahan T, Rehnqvist N (2002). Prognostic implications of autonomic function assessed by analyses of catecholamines and heart rate variability in stable angina pectoris. Heart.

[24] Bauer A, Kantelhardt JW, Barthel P, Schneider R, Makikallio T, Ulm K (2006). Deceleration capacity of heart rate as a predictor of mortality after myocardial infarction: cohort study. Lancet.

[25] Gomide Braga T, das Gracas Coelho de Souza M, Maranhao PA, Menezes M, Dellatorre-Teixeira L, Bouskela E, et al. Evaluation of heart rate variability and endothelial function 3 months after bariatric surgery. Obes Surg. 2020;30(6):2450–3. 10.1007/s11695-020-04397-4 31916132

[26] Brinkley DM, Ali OM, Zalawadiya SK, Wang TJ (2017). Vitamin D and heart failure. Curr Heart Fail Rep.

[27] Gotsman I, Shauer A, Zwas DR, Hellman Y, Keren A, Lotan C (2012). Vitamin D deficiency is a predictor of reduced survival in patients with heart failure; vitamin D supplementation improves outcome. Eur J Heart Fail.

[28] Plesner JL, Dahl M, Fonvig CE, Nielsen TRH, Kloppenborg JT, Pedersen O (2018). Obesity is associated with vitamin D deficiency in Danish children and adolescents. J Pediatr Endocrinol Metab.

[29] Kendrick J, Targher G, Smits G, Chonchol M (2009). 25-Hydroxyvitamin D deficiency is independently associated with cardiovascular disease in the Third National Health and Nutrition Examination Survey. Atherosclerosis.

[30] de Vries MA, van der Meulen N, van de Geijn GM, Klop B, van der Zwan EM, Prinzen L (2017). Effect of a single dose of vitamin D3 on postprandial arterial stiffness and inflammation in vitamin D-deficient women. J Clin Endocrinol Metab.

